# Risk estimates of mortality attributed to low concentrations of ambient fine particulate matter in the Canadian community health survey cohort

**DOI:** 10.1186/s12940-016-0111-6

**Published:** 2016-02-11

**Authors:** Lauren Pinault, Michael Tjepkema, Daniel L. Crouse, Scott Weichenthal, Aaron van Donkelaar, Randall V. Martin, Michael Brauer, Hong Chen, Richard T. Burnett

**Affiliations:** Health Analysis Division, Statistics Canada, 100 Tunney’s Pasture Driveway, Ottawa, ON K1A 0T6 Canada; New Brunswick Institute for Research, Data, and Training (NB-IRDT) and Department of Sociology, University of New Brunswick, Fredericton, NB PO Box 4400, E3B 5A3 Canada; Air Health Effects Science Division, Health Canada, 269 Laurier Avenue West, Ottawa, ON K1A 0K9 Canada; Department of Physics and Atmospheric Science, Dalhousie University, 6310 Coburg Road, Halifax, NS Canada; Harvard-Smithsonian Center for Astrophysics, 60 Garden St, Cambridge, MA 02138 USA; Faculty of Medicine/School of Population and Public Health, University of British Columbia, 2206 East Mall, Vancouver, BC V6T 1Z3 Canada; Public Health Ontario, Suite 300, 480 University Avenue, Toronto, ON M5G 1V2 Canada; Environmental Health Science and Research Bureau, Health Canada, 50 Columbine Driveway, Ottawa, ON Canada

**Keywords:** PM_2.5_, Fine particulate matter, Air pollution, Cardiovascular mortality, Respiratory mortality

## Abstract

**Background:**

Understanding the shape of the relationship between long-term exposure to ambient fine particulate matter (PM_2.5_) concentrations and health risks is critical for health impact and risk assessment. Studies evaluating the health risks of exposure to low concentrations of PM_2.5_ are limited. Further, many existing studies lack individual-level information on potentially important behavioural confounding factors.

**Methods:**

A prospective cohort study was conducted among a subset of participants in a cohort that linked respondents of the Canadian Community Health Survey to mortality (*n* = 299,500) with satellite-derived ambient PM2.5 estimates. Participants enrolled between 2000 and 2008 were followed to date of death or December 31, 2011. Cox proportional hazards models were used to estimate hazard ratios (HRs) for mortality attributed to PM_2.5_ exposure, adjusted for individual-level and contextual covariates, including smoking behaviour and body mass index (BMI).

**Results:**

Approximately 26,300 non-accidental deaths, of which 32.5 % were due to circulatory disease and 9.1 % were due to respiratory disease, occurred during the follow-up period. Ambient PM_2.5_ exposures were relatively low (mean = 6.3 μg/m^3^), yet each 10 μg/m^3^ increase in exposure was associated with increased risks of non-accidental (HR = 1.26; 95 % CI: 1.19-1.34), circulatory disease (HR = 1.19; 95 % CI: 1.07–1.31), and respiratory disease mortality (HR = 1.52; 95 % CI: 1.26–1.84) in fully adjusted models. Higher hazard ratios were observed for respiratory mortality among respondents who never smoked (HR = 1.97; 95 % CI: 1.24–3.13 vs. HR = 1.45; 95 % CI: 1.17–1.79 for ever smokers), and among obese (BMI ≥ 30) respondents (HR = 1.76; 95 % CI: 1.15-2.69 vs. HR = 1.41; 95 % CI: 1.04–1.91 for normal weight respondents), though differences between groups were not statistically significant. A threshold analysis for non-accidental mortality estimated a threshold concentration of 0 μg/m^3^ (+95 % CI = 4.5 μg/m^3^).

**Conclusions:**

Increased risks of non-accidental, circulatory, and respiratory mortality were observed even at very low concentrations of ambient PM_2.5_. HRs were generally greater than most literature values, and adjusting for behavioural covariates served to reduce HR estimates slightly.

**Electronic supplementary material:**

The online version of this article (doi:10.1186/s12940-016-0111-6) contains supplementary material, which is available to authorized users.

## Background

Ambient fine particulate air pollution (PM_2.5_) is known to contribute to cardiovascular and respiratory morbidity, and is recognized as an important contributor to global disease burden [1]. Recent estimates from Global Burden of Disease suggest that ambient air pollution was responsible for nearly 2.9 million deaths per year in 2013 [2]. While ambient PM_2.5_ concentrations in Canada are generally below national and international guidelines, analyses from the 1991 Canadian Census Health and Environment Cohort (CanCHEC) suggest that long-term exposure to PM_2.5_ in Canada (mean = 8.9 μg/m^3^) may contribute to non-accidental and cardiovascular mortality [3]. However, that study did not include individual-level information on potentially important confounding factors such as smoking and obesity and applied an indirect approach to adjust for these and other factors [3, 4]. Analysis of the United States Agricultural Health Study (AHS) cohort also supports an association between cardiovascular mortality and long-term exposure to low concentrations of ambient PM_2.5_ (mean = 9.2 μg/m^3^) [5]. Moreover, a recent meta-analysis of studies conducted in North America and internationally supports an association between long-term exposure to PM_2.5_ and mortality, with the strongest association observed for cardiovascular mortality [6].

The WHO PM_2.5_ guideline of 10 μg/m^3^ was based on the lower end of the exposure distribution in previous studies [1], though there are few studies that have evaluated concentration-response associations at very low exposures. The Global Burden of Disease 2010 study [1] developed a mortality risk model for PM_2.5_ over the global range of concentrations. This model incorporated a counterfactual uncertainty distribution, below which no excess risk was assumed, and was specified by a uniform distribution between 5.8 μg/m^3^ and 8.8 μg/m^3^. This uncertainty distribution was selected based on the lack of empirical evidence of any statistical association between ambient PM_2.5_ and mortality below their counterfactual distribution. These concentrations represent the 48.9^th^ and 79.9^th^ percentiles of the exposure distribution in this study, respectively. Therefore, it is of interest to examine the shape of the concentration-mortality association at these very low concentrations, as well as the statistical strength of evidence for such an association.

In this study, we examine the relationship between long-term exposure to ambient PM_2.5_ and non-accidental, respiratory, and cardiovascular mortality in the Canadian Community Health Survey (CCHS) cohort. Participants in this cross-sectional survey were enrolled across Canada between 2000 and 2008 and provided detailed individual-level information on potentially important confounding factors (e.g. smoking, obesity) that were not available for the previous analysis of PM_2.5_ and mortality in the CanCHEC study [3]. As such, the primary aim of this study was to examine the relationship between very low concentrations of PM_2.5_ (mean = 6.3 μg/m^3^) and different causes of mortality in Canada and the impacts of adjusting for potential confounding factors. Finally, an improved, finer-scale, satellite-derived exposure model for PM_2.5_ (i.e., a 1 km^2^ grid) was used to reduce exposure misclassification.

## Methods

### Data sources

The CCHS is a national, cross-sectional survey providing information about the health, behaviours, and health care use of the non-institutional Canadian population aged 12 or older. The survey excludes full-time members of the Canadian Armed Forces and residents of Indian reserves and certain remote areas. Exclusions represent less than 3 % of the target population of Canada [7]. The annual component of the CCHS was conducted every two years from 2000/01 to 2007, after which the survey was conducted on an annual basis. The CCHS response rates are as follows: 84.7 % in Cycle 1.1 (2000/01), 80.7 % in Cycle 2.1 (2003), 78.9 % in Cycle 3.1 (2005), 77.6 % in 2007, and 75.0 % in 2008 [7]. CCHS respondents were eligible for the CCHS-mortality cohort if they gave permission to share and link their information with other administrative datasets; 86.0 % of CCHS respondents agreed to the linkage.

The Canadian Mortality Database (CMDB) is a national database that contains all deaths registered in Canada since 1950. Deaths that occurred between January 1, 2000 and December 31, 2011 were eligible for linkage. The CMDB includes data on underlying cause of death and date of death.

The Historical Tax Summary File (HTSF) is a database of annual tax returns that represent all individuals who received a tax declaration in a given year. Tax years between 1996 and 2011 were eligible for linkage. The HTSF includes postal codes, names, and dates of death (if applicable).

### Linkage methodology

The creation of the CCHS-Mortality Cohort was conducted in two steps. First, using a probabilistic linkage methodology based on the Fellegi-Sunter theory of record linkage [8], eligible CCHS respondents were linked to the HTSF (using date of birth, sex, name, and postal code), in order to capture these variables and date of death, as reported on tax files between 1996 and 2011. Approximately 85 % of eligible CCHS respondents were linked to the HTSF. Alternative postal codes and names were captured through this initial linkage and were used in the subsequent linkage to the CMDB, to improve linkage results. Second, all eligible CCHS respondents (regardless of whether they were linked to the HTSF) were also linked to the CMDB (which included underlying cause of death), using standard probabilistic linkage techniques (as described above) and followed for mortality from cohort entry (i.e., date of CCHS interview) to December 31, 2011.

### Data preparation

A total of 457,300 eligible CCHS-mortality respondents were included, with 117,800 respondents in Cycle 1, 112,900 respondents in Cycle 2, 113,900 respondents in Cycle 3, and 112,700 respondents in 2007/08. CCHS respondents who were first linked to the HTSF had a greater probability to be linked to the CMDB since additional data in the HTSF (e.g., alternate postal codes, name, and date of death), were used in the probabilistic linkage. In order to reduce the probability of false-negative links, we excluded those CCHS respondents who were not linked to the HTSF (*n* = 69,300 respondents excluded) (Additional file 1).

Since the purpose of this analysis was to evaluate long-term effects of air pollution exposure, the study population was restricted to adults aged 25 to 90 years of age at enrollment (*n* = 72,000 respondents excluded). Adults older than 90 years of age were excluded from this study to ensure a sufficient sample size within all age strata. Similar to the CanCHEC study [3], immigrants living in Canada for less than 20 years (i.e., those who had arrived in Canada less than 20 years before the start date), were excluded from this study (*n* = 13,200 additional respondents excluded) for the following reasons. Immigrants are known to have better health and live longer than the Canadian-born population [9]. Immigrants also more frequently live in areas of greater ambient air pollution (unpublished data), and their exposure to air pollution prior to living in Canada is largely unknown. Cause-specific mortality analyses among recent immigrants were also not meaningful due to small sample sizes in the CCHS cohort (i.e., < 250 deaths). Therefore, the use of a larger cohort would be necessary to examine the health effects of air pollution on recent immigrant populations. Finally, we excluded an additional 3,400 respondents who were not linked to air pollution estimates since they live beyond the boundaries of the air pollution models (i.e., in the northern Territories) (Additional file 1). The final analytical sample was 299,500 respondents (note slight inconsistencies due to rounding). All research using human data was carried out at Statistics Canada in accordance with the *Statistics Act* to meet standards of privacy and confidentiality associated with the internal use of survey data. The record linkage project was approved by the Executive Management Board at Statistics Canada (ref. num. 003–2015).

The place of residence of respondents at the date of entry into the Cohort was mapped in Geographic Information Systems (ArcGIS v.10; ESRI 2010) through the use of Statistics Canada’s Postal Code Conversion File plus (PCCF+) V.6B, which assigns geographic coordinates to postal codes based on a population-weighted random allocation algorithm [10]. Respondent locations were then spatially linked to estimates from a surface layer of PM_2.5_ concentration derived by relating total column aerosol optical depth retrievals from the Moderate Resolution Imaging Spectroradiometer (MODIS) instrument to near-surface PM_2.5_ using the GEOS-Chem chemical transport model. Geographically weighted regression, which includes ground monitoring data and land use information, was subsequently applied to these estimates to produce average PM_2.5_ concentrations at a 0.01° × 0.01° (approximately 1 km^2^) resolution from 2004 to 2012 [11]. These models included coverage for nearly all of mainland North America. These estimates were extended to 1998 to 2003 using the inter-annual variation of Boys et al. (2014) [12], who inferred global PM_2.5_ trends at 0.1° × 0.1° resolution using satellites from 1998 to 2012. Average PM_2.5_ levels were strongly correlated with ground-level observations in North America (R^2^ = 0.82, slope = 0.97; *n* = 1440) [11]. Outliers that included PM_2.5_ values >20 ug/m^3^ were excluded from analysis (<1 % of respondents were excluded in this manner in any year). These outliers were likely due to inaccurate estimates of aerosol optical depth from satellite retrievals. For each year in the cohort, respondents were assigned a PM_2.5_ value corresponding to the mean of the three previous years to the follow-up year; therefore, exposure always preceded response. For example, for the follow-up year 2001, we assigned the mean PM_2.5_ estimates from 1998 to 2000.

### Covariates and statistical methods

Standard Cox proportional hazards models [13] were used for survival analysis of non-accidental and cause-specific mortality within the cohort, from the date of interview for the CCHS to either the date of death recorded in the CMDB or the final date of the linkage project (i.e., 31 December, 2011). All models were stratified by sex and age (5-year intervals). Socioeconomic covariates included: immigrant status, visible minority status, Aboriginal status, and marital status, educational attainment, income adequacy quintile, and employment status (Table 1). Visible minority status was defined as in the *Employment Equity Act*, as “persons, other than Aboriginal peoples, who are non-Caucasian in race or non-white in colour” [14]. Income adequacy quintiles were calculated based on the ratio of household income to the low-income cut-off for their household and community size. Low-income cut-offs represent families that spend more than 20 % of their income on food, shelter and clothing, and are adjusted for size of family and area of residence [14].

**Table 1:** Descriptive statistics of the study cohort and PM_2.5_ exposure, with Cox proportional HRs for each covariate

			95 % C.I.		PM_2.5_	
Covariate	Persons^+^	HR^ǂ^	Lower	Upper	Mean	SD
All	299,500	−	−	−	6.32	2.54
Sex						
Male	137,800	−	−	−	6.28	2.54
Female	161,700	−	−	−	6.36	2.54
Age group†						
25–34 years	52,500	−	−	−	6.39	2.54
35–44 years	59,400	−	−	−	6.29	2.50
45–54 years	58,100	−	−	−	6.21	2.51
55–64 years	54,900	−	−	−	6.20	2.51
65–74 years	41,700	−	−	−	6.41	2.58
75–90 years	32,900	−	−	−	6.58	2.64
Immigrant status						
Not an immigrant	270,300	1.000	−	−	6.19	2.50
Immigrant (in Canada ≥ 20 years)	28,800	*0.863	0.834	0.894	7.57	2.52
Visible minority status						
White	281,000	1.000	−	−	6.31	2.53
Visible minority	17,700	0.938	0.877	1.004	6.49	2.67
Aboriginal status						
Not Aboriginal	289,600	1.000	−	−	6.36	2.54
Aboriginal	9,200	*1.390	1.267	1.525	5.12	2.21
Marital status						
Married or common-law	183,500	1.000	−	−	6.09	2.46
Separated, divorced, widowed	69,500	*1.344	1.306	1.382	6.62	2.60
Single, never married	46,400	*1.512	1.446	1.581	6.82	2.63
Educational attainment						
Not completed high school	71,700	1.000	−	−	6.01	2.58
High school diploma	113,500	*0.829	0.806	0.852	6.25	2.50
Post-secondary diploma/certificate	64,900	*0.723	0.694	0.753	6.43	2.51
University degree	47,100	*0.581	0.552	0.611	6.83	2.51
Low income adequacy quintile						
1st quintile - lowest	56,200	1.000	−	−	6.53	2.64
2nd quintile	54,500	*0.787	0.762	0.813	6.37	2.58
3rd quintile	53,000	*0.662	0.637	0.689	6.37	2.52
4th quintile	53,300	*0.583	0.557	0.610	6.34	2.49
5th quintile - highest	56,700	*0.483	0.458	0.509	6.17	2.43
Employment status						
Employed	174,500	1.000	−	−	6.31	2.50
Not employed: looked for work‡	7,300	*1.522	1.319	1.757	6.20	2.61
Not employed: did not look for work‡	78,100	*1.818	1.732	1.908	6.25	2.55
Permanently unable to work	9,800	*4.533	4.274	4.808	6.43	2.64
Body Mass Index^a^						
Underweight (<18.5)	3,700	*2.140	1.989	2.303	6.76	2.60
Normal weight (18.5 - 25.0)	93,700	1.000	−	−	6.54	2.55
Overweight (25.0 - 30.0)	114,900	*0.804	0.781	0.828	6.29	2.52
Obese I (30.0 - 35.0)	54,700	*0.884	0.852	0.917	6.14	2.52
Obese II (>35.0)	24,200	*1.270	1.209	1.334	6.06	2.53
Fruit and vegetable consumption						
<5 servings per day	153,200	1.000	−	−	6.38	2.56
≥5 servings per day	101,100	*0.828	0.806	0.851	6.52	2.52
Smoking						
Never smoked	84,100	1.000	−	−	6.41	2.53
Former smoker	139,200	*1.284	1.244	1.324	6.26	2.51
Current daily or occasional smoker	75,900	*2.604	2.509	2.702	6.33	2.59
Alcohol						
Regular drinker (≥1 drink per month)	141,700	1.000	−	−	6.51	2.55
Occasional or former drinker	80,800	*1.394	1.356	1.433	6.25	2.59
Never drinker	11,000	*1.274	1.214	1.337	6.17	2.64
Ecological covariates^b^						
% recent immigrants (CD-DA)	−	*1.102	1.064	1.141	−	−
% recent immigrants (CD)	−	*0.713	0.680	0.747	−	−
% completed high school (CD-DA)	−	*0.928	0.919	0.938	−	−
% completed high school (CD)	−	*0.897	0.886	0.908	−	−
% in low income families (CD-DA)	−	*1.119	1.107	1.131	−	−
% in low income families (CD)	−	*1.100	1.070	1.131	−	−

+Numbers were rounded to the nearest 100 for confidentiality

ǂModels were stratified by age (5 year categories) and sex

*Significant HR (*p* < 0.05)

†At time of entry into the cohort

‡(Did not) look for work in past 4 weeks

^a^After adjusting for self-reporting bias in CCHS, as in [16]

^b^HRs provided for 10 % increase in population

Neighbourhood socioeconomic status, including both social and material deprivation, contributes to increased risk of mortality in Canadian cities, although the presence of immigrants can reduce mortality risk [15]. Ecological (contextual) covariates were derived from the long-form Canadian Census at the Census Division (CD) and Dissemination Area (DA) geographic scale, from the 2001 Census for respondents interviewed between 2000 and 2003, and the 2006 Census for respondents interviewed during or after 2004. Census Divisions are a subdivision of the provinces and territories that usually represent communities, regional districts, or several neighbouring municipalities, and range in size from several thousand to a few million persons [14]. Dissemination Areas are the smallest geographical unit used by the Census and are delineated based on population counts based on the previous census, to target a population of 400–700 persons [14]. There were 288 CDs and 54,623 DAs in Canada as of 2006 [14]. These contextual covariates were then linked to individual respondents through a common geographic identifier (i.e., a numeric code identifying the DA or CD). For each CD and DA, the proportion of recent immigrants (<5 years residency in Canada), educational attainment (the proportion of persons aged 15 years or older who had not graduated from high school) and low income (the proportion of persons below the low-income cut-off) were derived for both Census years [16]. The proportion of recent immigrants in a region may provide a health benefit in the form of social inclusion if the resident is a member of a unified community, though it also may represent social deprivation, since recent immigrants also include persons of very low SES upon arrival in Canada (e.g., refugees or temporary workers). The other two ecological covariates (educational attainment and low income) provide a more direct estimate of neighbourhood socioeconomic status. Although broader geographic scales such as Census Tracts (CTs) are more often used to derive neighbourhood contextual variables [16], CTs were not available for rural areas. Neighbourhood covariates were therefore calculated by taking the difference between CD and DA estimates. It was expected *a priori* that the ecological covariates would attenuate risk estimates as in previous work on CanCHEC [3].

In addition to the socioeconomic and ecological covariates, this study included four health status/behavioural covariates. Body Mass Index (BMI) was derived from the self-reported height and weight of respondents, and adjusted using correction factors that were developed for the CCHS to account for self-reporting bias in BMI data [17]. The International Standard Classification was used to categorize Body Mass Index [18], with obesity subdivided into two categories (i.e., BMI 30 – 34.9 and BMI ≥ 35) to further differentiate health risks among obese persons within the study. Smoking behaviour was categorized as never, former, or current smokers. Detailed data on smoking behaviour (e.g., number of cigarettes smoked per day) were available only for daily smokers (ca. 21.3 % of respondents) and were therefore not included. Fruit and vegetable daily consumption and alcohol consumption were also included, as in previous studies [19] (Table 1).

Survival models were examined in a sequential manner by adding all of the socioeconomic covariates in a single model, then adding in the ecological covariates to the socioeconomic models, and finally by adding the behavioural covariates to create fully adjusted models for non-accidental mortality (ICD-10 codes A-R) and mortality attributed to circulatory disease (ICD-10: I00–I99, with and without diabetes, E10–E14), including the subgroups of ischemic heart disease (ICD-10: I20–I25), and cerebrovascular disease (ICD-10: I60–I69). We also considered models for mortality due to respiratory disease (ICD-10: J00–J99), also including chronic obstructive pulmonary disease (ICD-10: J19–J46), and lung cancer (ICD-10: C33–C34). We also examined a model of socioeconomic and behavioural covariates, excluding ecological covariates. We added groups of variables in this manner to specifically examine the influence of including the behavioural variables to a model which included both socioeconomic and ecological variables, as were available in previous cohort studies in Canada [3]. Effect modification by sex, smoking behaviour (ever smoked vs. never smoked), BMI (obese: BMI ≥ 30 and obese II: BMI ≥ 35 vs. normal weight: BMI = 18.5–25), fruit and vegetable consumption (<5 servings vs. ≥ 5 servings), alcohol consumption (regular drinker vs. occasional/never/former drinker), and age (<75 years vs. ≥ 75 years) were also evaluated in separate Cox proportional hazards models, and Cochran’s Q-statistic heterogeneity tests were used to evaluate significant differences in HRs among groups [20]. These covariates were chosen for effect modification analysis due to known physiological differences between these groups of respondents, and interest in previous studies [6].

To examine the shape of the relationship between non-accidental mortality hazard ratio (HR) and air pollution exposure, we fitted spline-based HR curves using the smoothing method in the R package “smoothHR” on the fully adjusted model [21]. The package uses a combination of AIC and BIC to determine the optimal degrees of freedom to use in the model [21]. We also estimated the PM_2.5_ threshold concentration (T) by fitting Cox proportional hazards models to a series of newly defined PM_2.5_ based variables of the form: PM_2.5_ (T) = PM_2.5_ – T; if PM_i_ > T and 0 otherwise, for T = 1 to 10. Our estimate of T is the concentration corresponding to the largest (−2) log-likelihood value (−2LL) obtained from the Cox model. Ninety-five percent confidence intervals on T were based on changes in -2LL of 3.84 units.

All descriptive statistics reported from the survey were rounded to the nearest hundred for institutional confidentiality reasons.

## Results

**Fig. 1:**
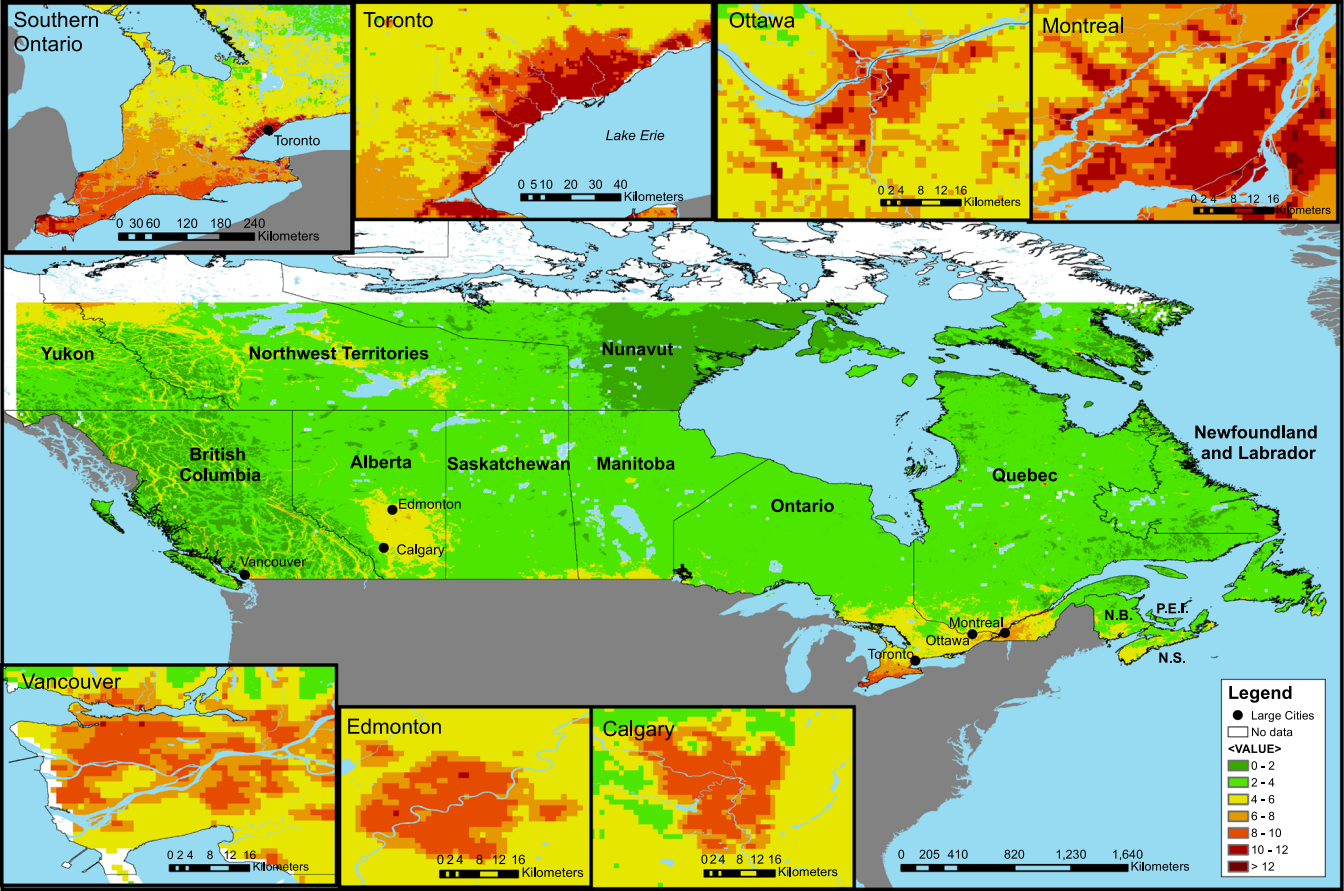
Map of mean PM_2.5_ estimates in Canada from 1998–2010 derived from satellite. imagery at 1 km^2^ resolution. Cities with populations greater than 1 million (in the metropolitan area) are indicated. All of these large city PM_2.5_ exposures were >8 ug/m^3^. Insets: detailed PM_2.5_ estimates in southern Ontario, Toronto, Ottawa, Montreal, Vancouver, Edmonton, and Calgary

A total of 299,500 respondents were included in the study after excluding respondents who were not linked to a tax file, respondents who were not within the 25 to 90 year age range and were not recent immigrants (i.e., < 20 years in Canada), and respondents who were not linked to air pollution estimates. Respondents were followed for mortality for up to 12 years after cohort entry (mean follow-up period (± SD) was 7.6 ± 2.7 years). The mean exposure (± SD) of respondents to PM_2.5_ estimated from the 3-year moving average was 6.3 ± 2.5 μg/m^3^. The PM_2.5_ person-year exposure percentiles within the final study cohort were: minimum: 1.0 μg/m^3^, 5^th^: 3.0 μg/m^3^, 25^th^: 4.2 μg/m^3^, median: 5.9 μg/m^3^, 75^th^: 8.3 μg/m^3^, 95^th^: 11.3 μg/m^3^, and maximum: 13.0 μg/m^3^. In large cities (metropolitan pop. > 1 million), PM_2.5_ estimates were generally greater than in surrounding areas, and there were areas of the downtown core exceeding 8 ug/m^3^ in all of these cities (Fig. 1). Mean PM_2.5_ exposure increased incrementally by decreasing income quintile and was highest for respondents in the poorest income quintile (Table 1). PM_2.5_ exposure was also greatest for the most highly educated respondents (Table 1). Obese respondents were exposed to less air pollution than those of increasingly lower weight classes, with the greatest exposure among respondents classified as underweight (Table 1). Hazard ratios for non-accidental mortality were calculated for all variables and ecological covariates (Table 1). Among ecological covariates for DAs and CDs, the proportion of recent immigrants, high school graduates and low income families were positively correlated with average PM_2.5_ air pollution exposure (Table 2). The proportion of recent immigrants was protective for mortality at the broader landscape level (i.e., the CT), though increased HRs at the neighbourhood scale (i.e., DAs) (Table 1). Associations between all combinations of the covariates are provided in Additional file 2.

**Table 2:** Descriptive statistics of ecological covariates derived from the 2001 and 2006 Census^a^

		Percentile						Correlation with mean PM_2.5_
Variable	Min	5th	25th	50th	75th	95th	Max	
Aggregated by Dissemination Area								
% recent immigrants	0.0	0.0	0.0	0.0	1.7	9.0	69.0	0.303
% completed high school	0.0	47.4	63.6	73.5	82.4	92.3	100.0	0.245
% in low income families	0.0	1.5	5.9	10.9	18.4	35.1	100.0	0.235
Aggregated by Census Division								
% recent immigrants	0.0	0.1	0.3	0.7	1.9	9.5	16.7	0.424
% completed high school	31.2	52.3	65.8	72.7	78.6	85.1	88.6	0.462
% in low income families	3.4	7.8	10.5	12.9	15.3	21.1	37.1	0.192

^a^Source: 2001 or 2006 Census data were chosen based on the closest year to the Cohort entry

Separate Cox proportional hazards models were run for all covariates in the fully adjusted models. Immigrant status, greater educational attainment, higher income, being overweight or obese (type I), and increasing consumption of fruits and vegetables were all associated with a lower risk of non-accidental mortality (Table 1). Aboriginal status, being unmarried, being underweight or obese (type II), not employed, smoking, and not regularly drinking alcohol were associated with a greater risk of non-accidental mortality (Table 1).

Covariates were added in a stepwise manner to a Cox proportional hazards model for non-accidental mortality to assess their contribution to the model (Table 3). In general, the addition of socioeconomic covariates improved the model fit, and resulted in a significantly increased HR from the unadjusted model (Table 3; Cochran’s Q = 4.29; *p* = 0.04). The additional of behavioural covariates to the socioeconomic model reduced HRs somewhat, though not significantly (Table 3; Cochran’s Q = 0.23, *p* = 0.63). The addition of ecological covariates to the socioeconomic model, particularly the percentage of recent immigrants and high school graduates, also improved model fit and significantly increased HRs (Table 3; Cochran’s Q = 27.30, *p* < 0.01). The addition of behavioural covariates to create a fully adjusted model also improved model fit, though the HRs declined non-significantly from the second adjusted model (Table 3; Cochran’s Q = 2.41, *p* = 0.12).

**Table 3:** Cox proportional HRs for non-accidental mortality^a^ in the cohort, with stepwise addition of covariates

		95 % CI		
	HR^b^	Lower	Upper	(−2) log l
Unadjusted	1.028	0.981	1.077	447,246
*SES covariates added separately*				
Immigrant status	*1.069	1.019	1.120	447,165
Visible minority status	1.031	0.984	1.080	447,237
Aboriginal status	1.035	0.988	1.085	447,217
Marital status	0.999	0.954	1.047	446,677
Educational attainment	*1.114	1.063	1.168	446,442
Income adequacy quintiles	1.031	0.985	1.081	446,127
Employment	1.032	0.985	1.081	445,050
All socioeconomic covariates	*1.103	1.052	1.157	443,829
*All SES + ecological covariates added separately*				
% recent immigrants	*1.253	1.190	1.320	440,157
% completed high school	*1.349	1.278	1.424	437,545
% low income	1.045	0.994	1.099	433,397
All SES + all ecological covariates	*1.345	1.270	1.424	433,080
*All SES + all ecological + behavioural covariates added separately*				
Smoking	*1.341	1.267	1.420	431,304
Alcohol consumption	*1.292	1.221	1.368	432,308
Fruit and vegetable consumption	*1.342	1.267	1.421	433,004
Body Mass Index	*1.345	1.270	1.424	432,338
All SES + all ecological + all behavioural covariates	*1.261	1.190	1.336	429,524

^a^Number of deaths = 26,300

^b^Models are stratified by age (5 year categories) and sex

*Significant HR (*p* < 0.05)

*SES* Socioeconomic

Table 4 presents the HRs and 95 % CI for Cox proportional hazard models for non-accidental mortality and mortality due to circulatory or respiratory causes. In the fully adjusted model, HR estimates for non-accidental mortality were 1.26 (95 % C.I.: 1.19–1.34) per 10 μg/m^3^ increase in ambient PM_2.5_. The strongest association was observed for respiratory disease mortality, with an HR of 1.52 (95 % C.I.: 1.26–1.84) per 10 μg/m^3^ increase in ambient PM_2.5_. In fully adjusted models, HRs were significantly greater than one for all causes of death except cerebrovascular disease and lung cancer, though the HRs were significant in the models that did not include behavioural covariates (Table 4). For all causes of death, HRs were greater in the fully adjusted model than in the unadjusted model, though were reduced after adding behavioural covariates (Table 4).

**Table 4:** Cox proportional HRs for mortality per 10 μg/m^3^ increase in ambient PM_2.5_ in the study cohort (*n* = 299,500)

		Unadjusted^+^			Adjusted: SES^†^			Adjusted: SES^†^ + behavioural cov.^§^			Adjusted: SES^†^ + ecological cov.^‡^			Adjusted: SES^†^ + ecological cov.^‡^ + behavioural cov.^§^		
			95 % CI			95 % CI			95 % CI			95 % CI			95 % CI	
Cause of mortality	Deaths	HR	To	From	HR	To	From	HR	To	From	HR	To	From	HR	To	From
Non-accidental^a^	26,300	1.028	0.981	1.077	*1.103	1.052	1.157	*1.085	1.034	1.139	*1.345	1.270	1.424	*1.261	1.190	1.336
Circulatory disease^b^	8,600	0.940	0.866	1.020	1.014	0.932	1.102	0.997	0.917	1.085	*1.297	1.174	1.434	*1.187	1.073	1.313
Circulatory-diabetes^c^	9,500	0.939	0.868	1.015	1.016	0.938	1.100	1.011	0.933	1.096	*1.313	1.194	1.444	*1.210	1.099	1.331
Ischemic heart d. ^d^	4,700	0.979	0.877	1.093	1.090	0.975	1.220	1.078	0.963	1.207	*1.408	1.232	1.610	*1.290	1.127	1.477
Cerebrovascular d. ^e^	1,500	1.064	0.879	1.288	1.082	0.890	1.316	1.063	0.872	1.295	*1.360	1.078	1.715	1.241	0.981	1.570
Respiratory disease^f^	2,400	1.133	0.970	1.324	*1.269	1.083	1.487	*1.214	1.034	1.425	*1.628	1.347	1.969	*1.522	1.257	1.843
COPD^g^	1,400	1.032	0.839	1.268	1.191	0.966	1.469	1.109	0.897	1.370	*1.480	1.150	1.903	*1.398	1.085	1.801
Lung cancer^h^	2,700	1.007	0.871	1.166	*1.170	1.008	1.357	1.088	0.937	1.263	*1.216	1.017	1.453	1.167	0.975	1.396

^+^Unadjusted and all adjusted models were stratified by age (5 year categories) and sex

^†^SES covariates: immigrant status, visible minority status, Aboriginal status, marital status, income adequacy quintile, educational attainment, and employment

^**§**^Behavioural covariates: smoking, alcohol consumption, fruit and vegetable consumption, and BMI

^‡^Ecological covariates: (CD-DA and CD) for % recent immigrants, % completed high school, and % low income household

*Significant HR, *p* < 0.05

^a^Includes ICD-10 codes A-R. ^b^Includes ICD-10 codes I00-I99. ^c^Includes ICD-10 codes I00-I99 and E10-E14. ^d^Includes ICD-10 codes I20-I25. ^e^Includes ICD-10 codes I60-I69. ^f^Includes ICD-10 codes J00-J99. ^g^Includes ICD-10 codes J19-J46. ^h^Includes ICD-10 codes C33-C34

The results of effect modification by sex, age, BMI (i.e., obese vs. normal weight), fruit and vegetable consumption (i.e., < 5 or ≥ 5 daily servings), smoking (i.e., ever smoked vs. never smoked) and alcohol consumption are presented in Table 5. In a fully adjusted model, the HR for non-accidental mortality among men was 1.34 (95 % C.I.: 1.24–1.46) per 10 μg/m^3^ increase in ambient PM_2.5_ and was significantly greater than that of women (Cochran’s Q; Table 5). The HRs for circulatory and respiratory disease mortality among men were also greater than among women, though the differences in HRs were not statistically significant (Cochran’s Q; Table 5). None of the other comparisons among groups were statistically significant (Table 5). However, the HR of respiratory disease mortality was particularly high among never smokers (HR: 1.97; 95 % CI: 1.23–3.13 per 10 μg/m^3^ increase in PM_2.5_) and among obese respondents (HR = 1.76, 95 % CI: 1.15–2.69 per 10 μg/m^3^ increase in PM_2.5_) (Table 5).

**Table 5:** Effect modification of Cox HRs^†^ by sex, age^ǂ^, smoking, obesity, and fruit/vegetable and alcohol consumption

			95 % CI				95 % CI		Cochran’s Q	
Cause of death	Deaths	HR	Lower	Upper	Deaths	HR	Lower	Upper	Q	p
	Females (*n* = 161,700)				Males (*n* = 137,800)					
Non-accidental	12,700	*1.181	1.088	1.282	13,000	*1.344	1.239	1.457	4.829	0.028
Circulatory	4,100	1.109	0.959	1.282	4,300	*1.268	1.101	1.459	1.687	0.194
Respiratory	1,100	1.323	0.998	1.754	1,300	*1.698	1.307	2.206	1.617	0.204
	<75 years old^ǂ^ (*n* = 266,600)				≥75 years old^ǂ^ (*n* = 32,900)					
Non-accidental	13,100	*1.248	1.151	1.353	12,600	*1.237	1.140	1.342	0.023	0.880
Circulatory	3,500	*1.239	1.058	1.450	4,900	1.100	0.965	1.254	1.295	0.255
Respiratory	1,000	*1.553	1.158	2.083	1,300	*1.461	1.136	1.878	0.096	0.757
	Ever Smoked (*n* = 215,100)				Never Smoked (*n* = 84,100)					
Non-accidental	19,400	*1.231	1.152	1.315	6,300	*1.397	1.242	1.571	3.381	0.066
Circulatory	6,000	*1.164	1.034	1.311	2,300	*1.287	1.060	1.563	0.749	0.387
Respiratory	1,900	*1.449	1.174	1.788	400	*1.966	1.236	3.129	1.376	0.241
	Obese I and II (*n* = 78,900)				Normal weight (*n* = 93,700)					
Non-accidental	6,200	*1.215	1.077	1.370	8,700	*1.264	1.147	1.394	0.250	0.617
Circulatory	2,100	1.110	0.903	1.364	2,700	1.125	0.945	1.339	0.009	0.922
Respiratory	500	*1.757	1.146	2.694	900	*1.408	1.041	1.905	0.688	0.407
	Obese II (*n* = 24,200)^b^				Normal weight (*n* = 93,700)					
Non-accidental	1,900	1.142	0.919	1.419	8,700	*1.264	1.147	1.394	0.698	0.403
Circulatory	700	0.888	0.609	1.294	2,700	1.125	0.945	1.339	1.247	0.264
	<5 fruit/veg servings (*n* = 153,200)				≥5 fruit/veg servings (*n* = 101,100)					
Non-accidental	12,900	*1.217	1.124	1.318	8,500	*1.199	1.087	1.322	0.054	0.817
Circulatory	4,100	1.098	0.954	1.263	2,900	*1.322	1.117	1.563	2.764	0.096
Respiratory	1,200	*1.421	1.091	1.852	700	*1.505	1.078	2.101	0.070	0.792
	Regular drinker (*n* = 141,700)				Not regular drinker^a^ (*n* = 91,800)					
Non-accidental	9,600	*1.280	1.168	1.403	13,300	*1.280	1.182	1.387	<0.001	1.000
Circulatory	2,900	*1.257	1.065	1.483	4,600	*1.201	1.048	1.376	0.174	0.677
Respiratory	800	*1.473	1.070	2.027	1,300	*1.449	1.120	1.875	0.006	0.938

^†^All models are stratified by age (5 year categories) and sex, and adjusted for the following covariates: immigrant status, visible minority status, Aboriginal status, marital status, educational attainment, income adequacy quintile, employment, body mass index, fruit and vegetable consumption, smoking, and alcohol. For each comparison, the stratum or covariate being compared was not included as a stratum/covariate in the model (i.e., smoking was not included as a covariate in the smoking comparison)

^ǂ^Age at entry into Cohort

+Cochran’s Q test for significant difference of HR between groups

*Significant HR (*p* < 0.05)

^a^Includes occasional, former, or never drinker

^b^Respiratory mortality not shown; mortality for obese II: n < 200

**Fig. 2:**
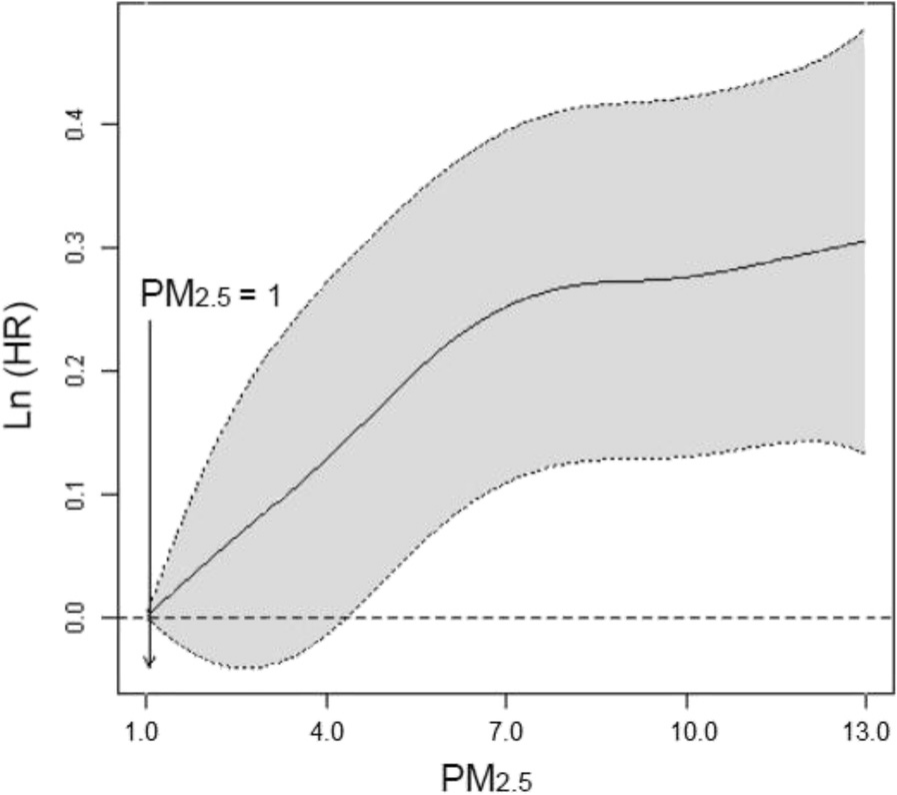
Nonparametric estimates of the dependence of non-accidental mortality on PM_2.5_ exposure among in-scope respondents in the CCHS-cohort linked to a PM_2.5_ dataset (log hazard ratio with 95 % confidence intervals). The model was stratified by age and sex, and adjusted for all covariates (Table 1). Model predictions were made up to the 99^th^ percentile of the PM_2.5_ exposure distribution

We fitted a nonparametric smoothing (spline) to examine the shape of the association between exposure and non-accidental mortality within the fully adjusted model. The relationship between the logarithm of the hazard function and PM_2.5_ is presented in Fig. 2 in addition to its 95 % confidence intervals. We specified a reference concentration of 1 μg/m^3^ which forces the predicted log-hazard function to equal 0 at the reference level. The smoothed curve generally increased with increasing concentration, however the confidence intervals are relatively wide making it difficult to speculate on a specific shape of the concentration-mortality association based on this graphical evidence. Our estimate of the threshold concentration was 0 μg/m^3^ with an upper 95 % CI value of 4.5 μg/m^3^.

## Discussion

Within our cohort, exposure to PM_2.5_ assigned at baseline was associated with an increased risk of non-accidental mortality and mortality due to circulatory and respiratory disease. Risks for all causes of death examined were greatest after adjusting for socioeconomic and ecological covariates, though were reduced after adjusting for smoking, alcohol consumption, BMI, and fruit/vegetable consumption. The largest hazard ratios per 10 μg/m^3^ increase in PM_2.5_ were observed for respiratory mortality compared to the other cause-specific estimates. Elevated risk was observed for respiratory mortality associated with air pollution among obese respondents and never-smokers, though the differences between these and reference groups were not statistically significant. We also examined the shape of the exposure-response curve, and although the lowest measured concentration of PM_2.5_ was 1 μg/m^3^, we found no lower threshold for response. Although this finding is potentially informative for burden assessment, it is worth noting that we did not distinguish between anthropogenic and natural sources of PM_2.5_ in this study.

This study adds to previous work in Canada, which has a generally lower mean PM_2.5_ exposure than other countries, by providing direct adjustments for behavioural covariates (i.e., smoking and obesity) that are known contributors to mortality. This study used similar methodology to a previous study in Canada, the Canadian Census Health and Environment Cohort (CanCHEC) [3], but was unable to directly evaluate the role of behavioural covariates. In general, our HR estimates for non-accidental mortality (HR = 1.26; 95 % CI: 1.19–1.34) were greater than those in CanCHEC (HR = 1.15; 95 % CI: 1.13–1.16; Cochran’s Q = 9.3, *p* < 0.01), though our estimates for circulatory death were similar (CCHS HR = 1.19; 95 % CI: 1.07–1.31; CanCHEC HR = 1.16; 95 % C.I.: 1.13–1.18; Cochran’s Q = 0.1, *p* = 0.8) (all units per 10 μg/m^3^ increase in PM_2.5_) [3].

The fact that we found stronger associations between mortality and PM_2.5_ here than were observed in the CanCHEC study [3] might be due to improvements in estimates of PM_2.5_. A new PM_2.5_ model developed at a much finer scale (1 km^2^ grid rather than 10 km^2^ grid) allowed respondents to be assigned more accurate, finer-scale estimates exposure to of PM_2.5_. This improved exposure model may have a particularly strong effect on respondents who live in mid-sized cities (e.g., Calgary, Edmonton) that would otherwise have been assigned a lower, regional (i.e., rural) average (Fig. 1). However, this improvement is expected to be limited somewhat by the limitations of location error in geocoding residences based on postal code, as well as respondent mobility throughout the study area, resulting in differences in personal exposure. Another strength of this study was that it assigned exposures to respondents in the three years preceding death, thereby ensuring that exposure always preceded health effects rather than being assigned concurrently. This method also takes long-term variation of exposure into account.

In our study, HR estimates increased after the addition of ecological covariates, which differs from the earlier CanCHEC study, in which the addition of ecological covariates served to decrease the HR estimates [3]. As described earlier, the ecological covariates used here were derived for smaller areas than in the CanCHEC study due to the absence of Census Tracts in rural areas. The methodological differences in deriving ecological covariates, particularly at a finer scale (i.e., assigning DA-level covariates rather than CT-level covariates), may also be one of the primary reasons why differences in HR estimates were observed between this study and CanCHEC, since fine scale covariates would be more spatially variable and covariates would more accurately reflect local socioeconomic conditions. Indeed, when the ecological covariates were removed from the Cox models of non-accidental mortality, the otherwise fully adjusted model provided an HR = 1.085 (Table 3), which is more consistent with the fully adjusted models in CanCHEC [3]. Ecological covariates included in this study were all positively correlated with PM_2.5_ (Table 2). Given the much greater PM_2.5_ exposure in urban environments, this association for recent immigrants and persons of high educational attainment is possibly due to a higher population of both in cities. The correlation with PM_2.5_ was weaker for the proportion of low income families, which was consistent with the similar proportions of regional-adjusted low-income families in rural and urban environments [22].

Our HRs for non-accidental mortality were greater than those reported for all-cause mortality in other, international studies that had considered the same behavioural covariates, though were generally similar when ecological covariates were excluded from our estimates [6, 23–25]. For example, the American Cancer Society study, which included 1.2 million adults in the United States, estimated an HR for all-cause mortality of 1.06 per 10 μg/m^3^ increase in PM_2.5_ (95 % C.I.: 1.02–1.11) after controlling for behavioural covariates, though that study did not include ecological covariates [23]. Similarly, a global, pooled meta-analysis estimated an all-cause mortality HR of 1.06 (95 % C.I.: 1.04–1.08) per 10 μg/m^3^ increase in PM_2.5_ [6]. However, our results with ecological covariates were not significantly different from those of a large meta-analysis of European studies, where the pooled HR estimate for natural-cause mortality adjusted for socioeconomic and behavioural covariates (though not large-scale socioeconomic covariates) was 1.09 per 5 μg/m^3^ increase in PM_2.5_ (95 % C.I.: 1.03–1.14) [26]. This estimate was not significantly lower than in our study (Cochran’s Q = 0.8, *p* = 0.4), where HR = 1.12 (95 % C.I.: 1.09–1.16) when scaled to a 5 μg/m^3^ increase in PM_2.5_.

Hazard ratio estimates for mortality due to circulatory disease (i.e., HR = 1.19) were generally consistent with those reported in the international literature, including the Harvard Six Cities study extended follow-up, which reported a HR of 1.28 per 10 μg/m^3^ increase in PM_2.5_ [6, 27], and a study in the U.K., which reported an HR of 1.05 per 1.9 μg/m^3^ increase in PM_2.5_ after adjustment for sex, age, BMI, and smoking (our study: HR = 1.03, 95 % CI: 1.01–1.05 when scaled to a 1.9 μg/m^3^ increase) [28]. However, our estimate was much greater than that reported from a study in Rome (HR = 1.06, 95 % CI: 1.04–1.08), which adjusted for some individual and area-based socioeconomic covariates [24], and the Dutch Environmental Longitudinal Study (DUELS), which reported an HR of 1.09 (95 % CI: 1.06–1.12) per 10 μg/m^3^ increase in PM_2.5_ [25].

Our hazard ratio estimates for respiratory disease (HR = 1.52) were generally greater than those in the literature, though literature estimates for HRs vary among studies. For example, one study in Rome that used area-based socioeconomic covariates identified a non-significant HR of 1.03 for respiratory disease [24]. On the other hand, the California Teachers Study identified an HR for respiratory mortality of 1.21 [29], and the Dutch cohort (DUELS) estimated an HR of 1.18 [25], which were similar to our HR estimate for respiratory mortality prior to adjustment for ecological covariates (HR = 1.21). Another study in the UK reported an HR of 1.17 (95 % CI: 1.12–1.22) per 1.9 μg/m^3^ increase in PM_2.5_ [28]. Our HR estimate after adjustment for ecological covariates was lower than this study (HR = 1.08, 95 % CI: 1.04–1.12) when scaled to a 1.9 μg/m^3^ increase in PM_2.5._

Our study also evaluated the role of effect modification by sex, age and behavioural covariates, and found a significantly greater HR estimate for non-accidental mortality among men than women. In a pooled European analysis of multiple cohorts, HRs were elevated among men but not women [26]. Our results are overall similar, although our generally greater HR estimates for non-accidental mortality might explain why HR was significant for both men and women. Men also had a greater HR than did women for circulatory disease mortality (though the differences were not significant), similar to the AHS cohort [5]. This finding was inconsistent with the results of a small (*n* = 3,239) cohort of white, non-smoking adults, where the relative risk of coronary heart disease mortality was elevated among women but not among men in a fully adjusted model [30]. Observed differences might be, at least in part, explained by relatively small cohort sizes.

Our HR estimates for non-accidental and circulatory mortality among obese and normal weight groups were not significantly different. Effect modification of cardiovascular mortality by obesity had previously been evaluated elsewhere in two all-female cohorts. One study identified a significantly greater HR with increasing BMI, with an HR for obese women of 1.35 (95 % C.I.: 1.12–1.64 per 10 μg/m^3^ increase in PM_2.5_) [31]. The other study did not test differences statistically among groups but did report an HR of 1.99 (95 % C.I.: 1.23–3.22 per 10 μg/m^3^ increase in PM_2.5_) for obese women [32]. The ACS also reported a greater HR among obese men [5]. In our study, obese respondents also had a high risk of respiratory mortality (HR = 1.76; 95 % C.I.: 1.15–2.69), though possibly due to a small number of deaths (*n* = 500), the HR estimate was not significantly different from the normal weight population.

In our study, persons who had never smoked had a qualitatively greater risk of non-accidental and circulatory mortality from fine particulate exposure than those who had smoked, though the difference between groups was non-significant (Table 5). This finding was consistent with the literature, where a marginally greater risk of cardiovascular mortality was observed among never smokers than among current or former smokers [6, 23, 33, 34]. In a Dutch cohort, respiratory mortality was qualitatively greater among current smokers than never smokers [34], a finding that was not consistent with our study.

There were several limitations with our study that may contribute to uncertainty in our estimates. The cohort was chosen because of the inclusion of various behavioural covariates, but it is generally much smaller than that of CanCHEC, which used the Census of population (i.e., 20 % of the population of Canada) [3]. Mean estimates of PM_2.5_ in Canada are generally lower than in other study countries [6], and the effect size is relatively small, requiring a large sample size to have adequate power for HR estimation. As a result, in our study the 95 % CIs were very wide in comparison to other studies [3], and we were also unable to adequately assess the shape of the concentration-response curves for other causes of death. It is also worth mentioning that our study relied on self-reported estimates for BMI and smoking. Although we were able to mathematically adjust BMI for self-reporting error based on measured BMI from another survey, it is possible that estimates of smoking may underrepresent actual smoking rates. Additionally, the follow-up period in our study was relatively short, particularly for respondents who entered the cohort in the final survey year (i.e., 2008, with a maximum of 4 years of follow-up). However, respondents entering the cohort in the first year of survey and who had remained in the cohort for the entire period were followed for a maximum of 12 years, which is comparable to the mean follow-up period (i.e., 12.6 years) in a review of other cohorts examining the same relationship [6]. The limitation of having a short follow-up period was mitigated somewhat by considering exposures that preceded the event.

In creating the cohort, 69,300 CCHS respondents were excluded since they were not linked to the HTSF (tax) file and were therefore not candidates for the probabilistic linkage. The excluded population were those who did not file a tax return, and the characteristics of this population differed somewhat from the cohort. In general, the excluded population was younger and had a lower educational attainment than the final cohort. Therefore, the cohort might be slightly biased towards higher educational attainment and those active in the labour market, though these same characteristics were used for adjustment in survival models.

Estimates of PM_2.5_ exposure were assigned at baseline at the person’s place of residence. Accuracy in geocoding residences was limited by the program PCCF+, which assigns residences to postal code representative points. The size of postal codes is relatively small (i.e. typically a few city blocks) in urban centres; therefore the PCCF+ program is highly accurate within these areas. However, estimates of PM_2.5_ exposure in rural areas are less likely to have been assigned accurately since postal code areas can be quite large. We performed a sensitivity analysis that considered only cohort members that lived within urban areas (i.e., Census Metropolitan Areas), and despite exposures being much greater in urban areas, results were not significantly different than those reported above (HR = 1.19, 95 % CI: 1.11–1.27, Cochran’s Q = 1.71, *p* = 0.19). Given the short follow-up period, we also did not assess mobility in this study, making the assumption that respondents did not move. By not assigning air pollution exposures based on changes to residential history, it is expected that there would be some degree of exposure misclassification associated with this limitation. A previous study using CanCHEC considered the assignment of exposures at baseline *vs*. considering mobility during the follow-up period on mortality risk attributed to PM_2.5_. In general, there was very little difference in HR estimates (i.e., HR = 1.03, 95 % CI: 1.02–1.03 from baseline exposure, *vs*. HR = 1.04, 95 % CI: 1.03–1.04 for exposure considering mobility) [35]. Although about 41 % of Canadians moved within the five-year period of 2001 to 2006 [36], the majority of moves were within cities or regions of similar PM_2.5_ exposures (not published). To assess this limitation, we ran a sensitivity analysis where we included only persons who had at least 3 years of residence in the same postal code. HRs for non-accidental mortality were similar to those for the entire cohort (HR = 1.28, 95 % CI: 1.19–1.37).

Finally, the cohort was developed based on a probabilistic linkage methodology to assign deaths to CCHS members. We attempted to reduce the potential for linkage error by limiting our cohort to persons linked to a tax file, since mortality rates among cohort members not linked to a tax file were substantially lower due to fewer elements of respondent data that could be used for linkage.

## Conclusions

In general, this study documented an association between non-accidental, circulatory, and respiratory mortality and fine particular matter in a cohort adjusted for socioeconomic, ecological, and behavioural covariates and exposed to a relatively low exposure distribution (mean = 6.3 μg/m^3^). Although our CI were wide in the concentration-response curve, an increased risk of mortality was observed even at very low concentrations of PM_2.5_ (Fig. 2), at values lower than the WHO guideline of 10 μg/m^3^ [2]. Further studies on a larger cohort are needed to evaluate the shape of the concentration-response curve at these lower concentrations of PM_2.5_. We also updated the results of previous Canadian studies by using an improved, finer-scale exposure model to assign PM_2.5_ estimates to cohort members, which may have, in part, caused observed increases in HR estimates relative to CanCHEC [3]. Finally, this study indicates that the addition of fine-scale behavioural covariates serves to reduce the HR estimates compared to the otherwise fully adjusted survival models.

## Additional files

Additional file 1:Figure S1. Selection of Study Cohort.(PDF 88 kb) Additional file 2: Table S1. Comparison of all variables (Pearson's correlation or ANOVA/T-Test). (XLSX 22 kb)

## Abbreviations

AHS: United States Agricultural Health Study
BMI: Body Mass Index
CanCHEC: Canadian Census Health and Environment Cohort
CCHS: Canadian Community Health Survey
CD: Census Division
CI: confidence interval
CMDB: Canadian Mortality Database
CT: census tract
DA: dissemination area
HR: hazard ratio
HTSF: Historical Tax Summary File
-2LL: (−2) Log-likelihood ratio
MODIS: moderate resolution imaging spectroradiometer
PCCF+: Postal Code Conversion File Plus
PM_2.5_: Fine particulate matter
WHO: World Health Organization

## References

[1] Lin SS, Vos T, Flaxman AD, Danaei G, Shibuya K, Adair-Rohani H (2012). A comparative risk assessment of burden of disease and injury attributable to 67 risk factors and risk factor clusters in 21 regions, 1990–2010: A systematic analysis for the Global Burden of Disease Study 2010. Lancet.

[2] GBD 2013 Risk Factors Collaborators. Global, regional and national comparative risk assessment of 79 behavioural, environmental/occupational and metabolic risks or clusters of risks in 188 countries 1990–2013: a systematic analysis for the GBD 2013. Lancet. 2015; in press. 10.1016/S0140-6736(15)00128-2 PMC4685753 26364544

[3] Crouse DL, Peters PA, van Donkelaar A, Goldberg MS, Villeneuve PJ, Brion O (2012). Risk of nonaccidental and cardiovascular mortality in relation to long-term exposure to low concentrations of fine particulate matter: A Canadian national-level cohort study. Environ Health Persp.

[4] Shin HH, Cakmak S, Brian O, Villeneuve P, Turner MC, Goldberg MS (2014). Indirect adjustment for multiple missing variables applicable to environmental epidemiology. Environ Res.

[5] Weichenthal S, Villeneuve PJ, Burnett RT, van Donkelaar A, Martin RV, Jones RR (2014). Long-term exposure to fine particulate matter: association with nonaccidental and cardiovascular mortality in the Agricultural Health Study Cohort. Environ Health Persp.

[6] Hoek G, Krishnan RM, Beelen R, Peters A, Ostro B, Brunekreef B (2013). Long-term air pollution exposure and cardio-respiratory mortality: a review. Environ Health.

[7] Canada S (2009). Canadian Community Health Survey (CCHS) annual component: User guide, 2007–2008 microdata files.

[8] Fellegi IP, Sunter AB (1969). A theory for record linkage. J Am Stat Assoc.

[9] Wilkins R, Tjepkema M, Mustard C, Choinière R (2008). The Canadian census mortality follow-up study, 1991 through 2001. Health Rep.

[10] Wilkins R, Peters PA. PCCF + Version 5K* User’s Guide. Automated geographic coding based on the Statistics Canada Postal Code Conversion Files including Postal Codes through May 2011. Statistics Canada: Ottawa, ON, Canada, 2012. Catalogue no. 82F0086-XDB.

[11] van Donkelaar A, Martin RV, Spurr RJD, Burnett RT. High-resolution satellite-derived PM_2.5_ from optimal estimation and geographically weighted regression over North America. Environ Sci Technol. 2015; in press (doi:10.1021/acs.est.5b02076). 10.1021/acs.est.5b02076 26261937

[12] Boys BL, Martin RV, van Donkelaar A, MacDonell RJ, Hsu NC, Cooper MJ (2014). Fifteen-year global time series of satellite-derived fine particulate matter. Environ Sci Technol.

[13] Cox DR (1972). Regression models and life tables. J Royal Stat Soc B.

[14] Statistics Canada. Census Dictionary 2006. Statistics Canada: Ottawa, ON, Canada, 2010. Catalogue no. 92-566-X.

[15] Ross NA, Oliver LN, Villeneuve PJ (2013). The contribution of neighbourhood material and social deprivation to survival: a 22-year follow-up of more than 500,000 Canadians. Int J Environ Res Public Health.

[16] Crouse DL, Peters PA, Villeneuve PJ, Proux M-O, Shin HH, Goldberg MS (2014). Within- and between-city contrasts in nitrogen dioxide and mortality in 10 Canadian cities; a subset of the Canadian Census Health and Environment Cohort (CanCHEC). J Expo Sci Env Epi.

[17] Connor Gorber S, Shields M, Tremblay MS, McDowell I (2008). The feasibility of establishing correction factors to adjust self-reported estimates of obesity in the Canadian Community Health Survey. Health Rep.

[18] World Health Organization. BMI Classification: The international classification of adult underweight, overweight, and obesity according to BMI. http://apps.who.int/bmi/index.jsp?introPage=intro_3.html (2006). Accessed 28 Jul 2015.

[19] Chen H, Burnett RT, Kwong JC, Villeneuve PJ, Goldberg MS, Brook RD (2013). Spatial associations between ambient fine particulate matter and incident hypertension. Circulation.

[20] Conover W (1999). Practical Nonparametric Statistics.

[21] Meira-Machado L, Cadarso-Suárez C, Gude F, Araújo A. smoothHR: An R package for pointwise nonparametric estimation of hazard ratio curves of continuous predictors. Comput Math Methods Med. 2013; doi:10.1155/2013/745742. 10.1155/2013/745742 PMC3876718 24454541

[22] Fortin, M. A comparison of rural and urban workers living in low-income. Rural and Small Town Canada Analysis Bulletin (Statistics Canada). 2008;7:1–18. Cat. no. 21-006-XIE.

[23] Pope CA, Burnett RT, Thun MJ, Calle EE, Krewski D, Ito K (2002). Lung cancer, cardiopulmonary mortality and long-term exposure to fine particulate air pollution. JAMA.

[24] Cesaroni G, Badaloni C, Gariazzo C, Staffogia M, Sozzi R, Davoli M (2013). Long-term exposure to urban air pollution and mortality in a cohort of more than a million adults in Rome. Environ Health Perspect.

[25] Fischer PH, Marra M, Ameling CB, Hoek G, Beelen R, de Hoogh K, et al. Air pollution and mortality in seven million adults: The Dutch Environmental Longitudinal Study (DUELS). Environ Health Perspect. 2015; doi: 10.1289/ehp.1408254. 10.1289/ehp.1408254 PMC4492265 25760672

[26] Beelen R, Raaschou-Nielsen O, Stafoggia M, Jovanovic Andersen Z, Weinmayr G, Hoffmann B (2014). Effects of long-term exposure to air pollution on natural-cause mortality: an analysis of 22 European cohorts within the multicentre ESCAPE project. Lancet.

[27] Laden F, Schwartz J, Speizer FE, Dockery DW (2006). Reduction in fine particulate air pollution and mortality: extended follow-up of the Harvard Six Cities Study. Am J Resp Crit Care.

[28] Carey IM, Atkinson RW, Kent AJ, van Staa T, Cook DG, Anderson HR (2013). Mortality associations with long-term exposure to outdoor air pollution in a national English cohort. Am J Respir Crit Care Med.

[29] Ostro B, Lipsett M, Reynolds P, Goldberg D, Hertz A, Garcia C (2010). Long term exposure to constituents of fine particulate air pollution and mortality: results from the California teachers study. Environ Health Persp.

[30] Chen LH, Knutsen SF, Shavlik D, Beeson WL, Petersen F, Ghamsary M (2005). The association between fatal coronary heart disease and ambient particulate air pollution: are females at greater risk?. Environ Health Persp.

[31] Miller KA, Siscovick DS, Sheppard L, Shepherd K, Sullivan JH, Anderson GL (2007). Long-term exposure to air pollution and incidence of cardiovascular events in women. New Engl J Med.

[32] Puett RC, Schwartz J, Hart JE, Yanosky JD, Speizer FE, Suh H (2008). Chronic particulate exposure, mortality, and coronary heart disease in the nurses’ health study. Am J Epidemiol.

[33] Krewski D, Jerrett M, Burnett RT, Ma R, Hughes E, Shi Y (2009). 2009. Extended follow-up and spatial analysis of the American Cancer Society study linking particulate air pollution and mortality. Res Rep Health Eff Inst.

[34] Beelen R, Hoek G, van Den Brandt PA, Goldbohm RA, Fisher P, Shouten LJ (2008). Long-term effects of traffic-related air pollution on mortality in a Dutch cohort (NLCS-AIR study). Environ Health Persp.

[35] Crouse DL, Peters PA, Hystad P, Brook JR, van Donkelaar A, Martin RV (2015). Ambient PM_2.5_, O_3_, and NO_2_ exposures and associations with mortality over 16 years of follow-up in the Canadian Census Health and Environment Cohort (CanCHEC). Environ Health Persp.

[36] Statistics Canada. 2006 Census data products: 2006 census trends. http://www12.statcan.ca/census-recensement/2006/dp-pd/92-596/P2-2.cfm?Lang=eng&T=CSD&LINE_ID=701&TOPIC_ID=700 (2010). Accessed 12 Dec 2015.

