# Supplementary material for: Risk estimates of mortality attributed to low concentrations of ambient fine particulate matter in the Canadian community health survey cohort
Source: Environ Health. 2016 Feb 11;15:18. doi: 10.1186/s12940-016-0111-6 (PMC4750218; doi:10.1186/s12940-016-0111-6)
Supplement: Additional file 1:Figure S1. — Selection of Study Cohort.(PDF 88 kb) [file 12940_2016_111_MOESM1_ESM.pdf]

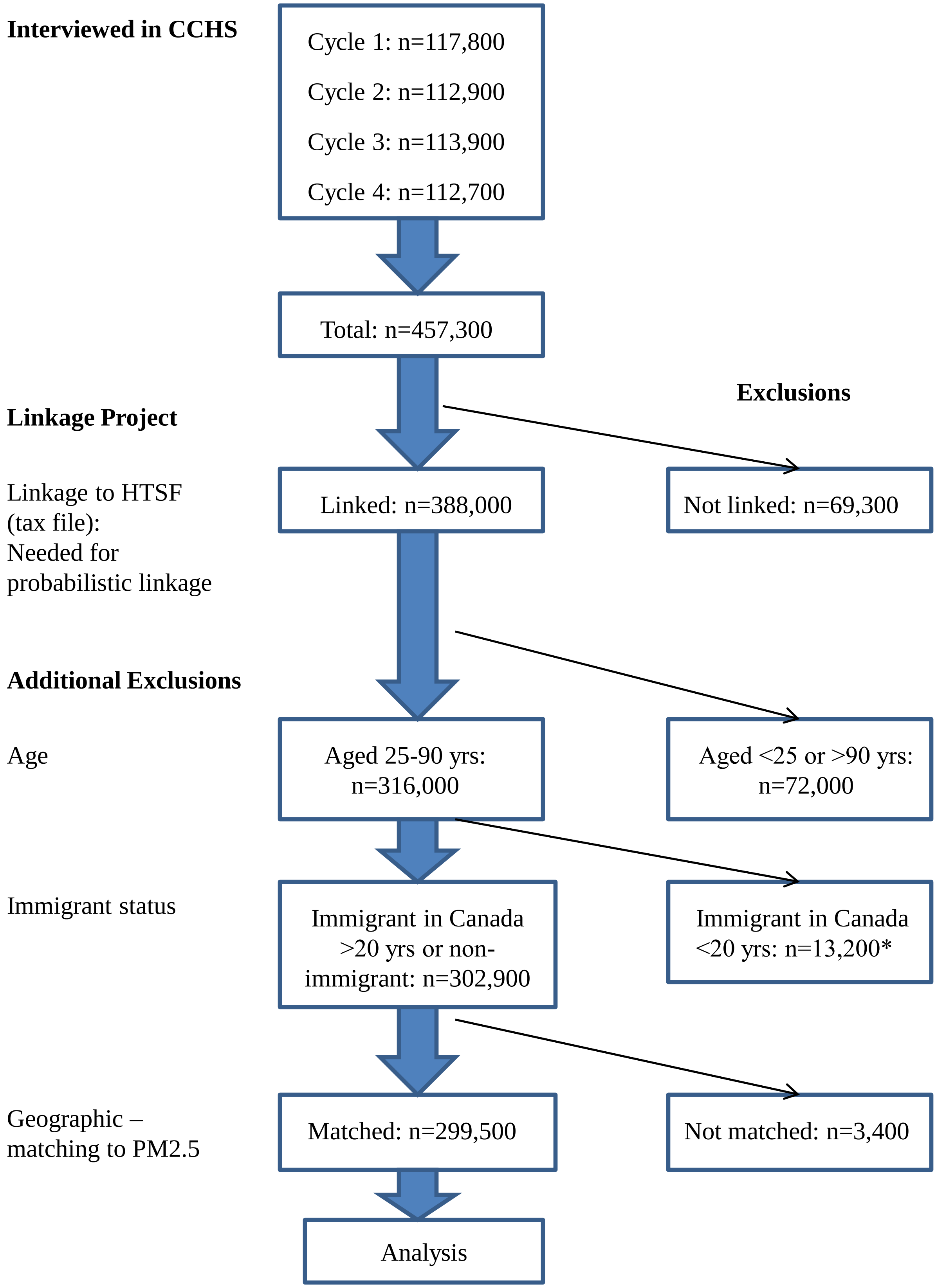

*mathematical inconsistency due to rounding.
